# IL-33 regulates adipogenesis via Wnt/β-catenin/PPAR-γ signaling pathway in preadipocytes

**DOI:** 10.1186/s12967-024-05180-0

**Published:** 2024-04-17

**Authors:** Danning Xu, Siqi Zhuang, Hongzhi Chen, Mengjie Jiang, Ping Jiang, Qian Wang, Xuemei Wang, Ruohong Chen, Haoneng Tang, Lingli Tang

**Affiliations:** 1 Department of Laboratory Medicine, The Second Xiangya Hospital, Central South University, Changsha, Hunan China; 2 National Clinical Research Center for Metabolic Disease, Key Laboratory of Diabetes Immunology, Ministry of Education, Metabolic Syndrome Research Center, and Department of Metabolism & Endocrinology, The Second Xiangya Hospital, Central South University, Changsha, Hunan China

**Keywords:** Obesity, Adipogenesis, Interleukin-33, 3T3-L1 cells, PPAR gamma, Wnt/β-catenin

## Abstract

Interleukin-33 (IL-33), an emerging cytokine within the IL-1 family, assumes a pivotal function in the control of obesity. However, the specific mechanism of its regulation of obesity formation remains unclear. In this study, we found that the expression level of IL-33 increased in visceral adipose tissue in mice fed with a high-fat diet (HFD) compared with that in mice fed with a normal diet (ND). In vitro, we also found the expression level of IL-33 was upregulated during the adipogenesis of 3T3-L1 cells. Functional test results showed that knockdown of IL-33 in 3T3-L1 cells differentiation could promote the accumulation of lipid droplets, the content of triglyceride and the expression of adipogenic–related genes (i.e. PPAR-γ, C/EBPα, FABP4, LPL, Adipoq and CD36). In contrast, overexpression of IL-33 inhibits adipogenic differentiation. Meanwhile, the above tests were repeated after over-differentiation of 3T3-L1 cells induced by oleic acid, and the results showed that IL-33 played a more significant role in the regulation of adipogenesis. To explore the mechanism, transcriptome sequencing was performed and results showed that IL-33 regulated the PPAR signaling pathway in 3T3-L1 cells. Further, Western blot and confocal microscopy showed that the inhibition of IL-33 could promote PPAR-γ expression by inhibiting the Wnt/β-catenin signal in 3T3-L1 cells. This study demonstrated that IL-33 was an important regulator of preadipocyte differentiation and inhibited adipogenesis by regulating the Wnt/β-catenin/PPAR-γ signaling pathway, which provided a new insight for further research on IL-33 as a new intervention target for metabolic disorders.

**Supplementary Information:**

The online version contains supplementary material available at 10.1186/s12967-024-05180-0.

## Introduction

Obesity is one of the biggest threats to worldwide public health in the 21st century. Obesity regulation is a complex process involving numerous cytokines [1, 2]. Unfortunately, much more researches are needed to explore the exact mechanisms underlying the genesis of obesity.

IL-33 is a newly discovered tissue-derived nuclear cytokine that belongs to the IL-1 family and is located in the cell nucleus [3]. Some studies have found that IL-33 can improve obesity, reduce inflammation in adipose tissue, and reduce glucose metabolism disorders [4, 5]. These researches confirmed the inhibitory effect of exogenous IL-33 on adipogenesis [6, 7]; despite of these, the results of endogenous IL-33 were contradictory [8, 9]. Previous studies reported that the expression level of IL-33 was significantly increased in the omental adipose tissue of severely obese patients and subcutaneous adipose tissue of diet-induced obese mice, but not in the adipose tissue of db/db mice with leptin receptor deficiency [10]. Xu-Yun Zhao et al. also found that IL-33 mRNA and protein expression levels were significantly increased in the adipose tissue of epididymis of obese mice induced by high fat diet [8]. However, T. Mahlakoiv et al. demonstrated that IL-33 expression was significantly decreased in adipose stem cells and progenitor cells of obese mice induced by high fat diet [11]. Therefore, the impact of endogenous IL-33 on obesity remains veiled. Moreover, it was also confirmed by our previous work that IL-33 levels were clinically associated with obesity phenotypes [12], which may be due to the complex components of adipose tissue as speculated based on aforementioned studies.

In vivo, adipose mesenchymal stem cells differentiate into preadipocytes, and these preadipocytes then proliferate and differentiate into adipocytes [13]. The process of differentiation of preadipocytes into adipocytes in the body is also known as “adipogenesis” and is the key step leading to fat accumulation and obesity [13]. Although adipocytes and preadipocytes do express IL-33 [14], the specific role of IL-33 in the adipogenesis of adipocytes has not been revealed.

Peroxisome proliferator-activated receptor-γ (PPAR-γ) is a crucial transcription factor that mediates the complex process of converting preadipocytes into mature adipocytes [15]. Researches have demonstrated that the classic Wnt/β-catenin pathway blocks the adipogenesis of preadipocytes by regulating the expression levels of key lipogenic genes such as PPAR-γ [16, 17]. Previous studies indicated that IL-33 inhibited the osteogenic differentiation of periodontal and pulp stem cells by acting on the Wnt/β-catenin pathway [18]. Therefore, whether IL-33 regulates the adipogenesis of preadipocytes via the aforementioned mechanisms remains to be explored.

In this study, we first explored the expression pattern of IL-33 by constructing high-fat diet (HFD)-induced mice and the adipogenesis cell model. Then, we explored the regulatory role of IL-33 secreted by adipocytes on adipogenesis through lentivirus knockdown or overexpression of IL-33. These findings might provide a basis for further understanding of the key regulatory role of IL-33 in adipogenesis and obesity, as well as potential clinical treatment strategies.

## Materials and methods

### Experimental animals

All animal experiments were performed according to the procedures approved by the Second Xiangya Hospital of Animal Care and Use Committee. Male C57BL/6J mice aged 6 weeks were purchased from Changsha Tianqin Biological Technology Co., Ltd. weighing between 17 and 22 g. They had unrestricted access to food and water while living at a temperature of 22 °C ± 2 °C, relative humidity of 50% ±10%, and a 12-hour light/dark cycle. After one week of acclimatization, the mice were separated into groups of similar weight using a random allocation method. Subsequently, the mice were administered either a standard diet (referred to as the normal diet or ND) including 10% fat (Xietong Pharmaceutical Bio-engineering Co., Ltd, D12450J) as the control group, or a high-fat diet (referred to as the HFD) comprising 60% fat (Xietong Pharmaceutical Bio-engineering Co., Ltd, D12492). The body weights of the mice were recorded at the same time every week. After 1, 2, 4 and 8 weeks of feeding, six mice from the control group and six mice in the high-fat feeding group were selected each time. The mice were killed, and their limbs were locked in the supine posture. The hair on their abdomen was removed by shaving and thereafter cleansed with alcohol. Moreover, a transverse incision was made on the abdomen skin to expose the underlying subcutaneous fat. The serum and the mesenteric adipose tissue were collected, packed, and kept at a temperature of − 80 °C for the follow-up experiments.

### Cell culture and treatment

The 3T3-L1 cell line was obtained from Guangzhou Saiku Biotechnology Co., Ltd. and maintained in Dulbecco’s Modified Eagle Medium (DMEM) (Gibco, C11995500BT) supplemented with 10% fetal calf serum (Bioexplorer, BS1612-109) and 1% penicillin/streptomycin (Biosharp, BL505A) at a temperature of 37 °C in a 5% CO_2_ environment. For differentiating these 3T3-L1 preadipocytes into adipocytes, 2 × 10^6^ cells were seeded into 6-well culture plates. After 72 h of incubation in the complete medium, the cells reached 100% confluence (day 0, D0). For conventional adipogenic differentiation (conventionally induced), DMEM with 10% fetal calf serum, 1 µM dexamethasone (Sigma-Aldrich, D4902), 5 µg/mL insulin (Novo Nordisk, S20191007), and 0.5 µM 3 isobutyl 1 methylxanthine (Sigma-Aldrich, I7018) were used to promote differentiation for 3 days (D0-D3). A differentiation medium was then utilized for another 3 days, consisting of DMEM with 10% FBS and 5 µg/mL insulin (D3-D6). Then, the differentiation medium was added again for another 2 days (D6-D8). On day 8, the medium was replaced with the regular medium (DMEM supplemented with 10% fetal calf serum) every other day.

In the hyper-adipogenic differentiation group (OA induced), the same degree of confluence was achieved on day0 as in the conventionally induced group. To ensure that oleic acid was constantly present during the lipid induction process, 250 μM oleic acid was then added to the induction reagent (as the same composition of the conventionally induced group described above)on days 0, 3, 6, and 8 of differentiation.

3T3-L1 preadipocytes were treated with 30 ng/mL recombinant IL-33 (Huamei, CSB-AP003411MO) during adipogenesis to examine the effect of IL-33 on adipogenesis. Then, the cells were collected for further experiments. For agonist experiments, we treated the cells with Wnt agonist 1 (10µM) (Selleck Chemicals, S8178) during differentiation. Wnt agonist 1 is a cell-permeable activator of the Wnt signaling pathway that induces β-catenin- and TCF-dependent transcriptional activity.

### Oleic acid preparation and treatment

Firstly, oleic acid (Sigma-Aldrich, O1383) was added into the NaOH solution to create the 100nM sodium oleate storage solution, which was incubated at 70℃ for 30 min. Then sodium oleate was slowly added to 10% BSA (Sigma-Aldrich, A1933) solution without free fatty acids, and was shaken at 55℃ for 1 h to obtain a 4.36mM storage solution. Finally, the oleic acid solution bound to albumin was filtered through a 0.2 μm syringe filter and stored at -20℃. Oleic acid (OA) was added during adipogenesis of 3T3-L1 cells, so that the working concentration was 250 μM.

### Cell transfection

IL-33-knockdown lentivirus (shIL-33) and IL-33-overexpression lentivirus (LV-IL-33) were packaged and supplied by GeneChem (Shanghai, China). The sequences were as follows: siIL-33: 5’CGTCCTCGGACTTTGTTTCATT-3’; and siNC: 5’TTCTCCGAACGTGTCACGT-3’. A total of 5 × 10^4^ 3T3-L1 cells were inoculated into each well of 6-well plates 24 h prior to lentiviral transfection, and cultured at 37 °C with 5% CO_2_. A 50 MOI was utilized for lentiviral transfection. The cells were incubated at 37 °C for 48 h before being used in the ensuing experiments.

### Quantitative polymerase chain reaction

The total RNA extraction was performed using the TRIzol reagent (Sangon Biotech, B511321) following the guidelines provided by the manufacturer. Total RNA was reverse transcribed into complementary DNA (cDNA) using the PrimeScript RT Reagent kit (TaKaRa Bio, RR047B), in accordance with the manufacturer’s instructions. Following the manufacturer’s instructions, quantitative polymerase chain reaction (qPCR) was then carried out using SYBR Green Premix Ex Taq II (TaKaRa Bio, RR820A). β-actin was used as the internal control. The primer sequences used in this study are depicted in Supplementary Material Table 1. qPCR was performed on a Roche LightCycler 96 PCR Detection System (Roche, Switzerland) following the manufacturer’s protocols. The thermocycling protocol consisted of an initial predenaturation step at 95 °C for 30 s, followed by 50 cycles of denaturation at 95 °C for 5 s, annealing at 60 °C for 30 s, and finally a dissociation program. The 2^ΔΔCq^ was calculated for relative quantification for each sample.

### Western blot analysis

Following the aforementioned treatment, the cells were harvested utilizing RIPA lysate (Thermo Fisher Scientific, 89900). The protein concentration was determined using the BCA assay(Thermo Fisher Scientific, No.23225). The proteins were resolved using 10–15% SDS gels (Epizyme Biomedical Technology, PG113) with equal quantities of 20 µg for each lane. Following resolution, the proteins were transferred onto PVDF membranes (Millipore, ISEQ00010). The membrane was then blocked with 5% skim milk (Beyotime, P0216) at room temperature for 1 h. Then the membranes were incubated with specific antibodies against the following proteins: IL-33 (Abcam, ab17831, 1:1000), H3 (Abcam, ab176842, 1:1000), GSK3β (Cell Signaling Technology, #12456, 1:1000), p-GSK3β (Ser9) (Cell Signaling Technology, #9323, 1:1000), LRP6 (Cell Signaling Technology, #33395, 1:1000), PPAR-γ (Cell Signaling Technology, #2435, 1:1000), β-catenin (Cell Signaling Technology, #8480, 1:1000), cyclin D1 (Cell Signaling Technology, #2978, 1:1000) and β-actin (Origene, CAT#TA811000, 1:2000) at 4 °C overnight. Then, the membrane was rinsed and incubated with HRP-conjugated secondary antibodies (Beyotime, A0208 and A0216, 1:5000) at room temperature for 1 h. Protein signals are visualized using chemiluminescent reagents (Millipore, WBLUC0500). Finally, densitometry analysis was used to determine the level of protein expression (ImageJ 1.46r; National Institutes of Health).

### Measurement of triglyceride content

Triglyceride (TG) contents of 3T3-L1 cells were measured with a TG assay reagent kit (single agent GPO-PAP method, Jiancheng, Nanjing, China, A110-1-1) in accordance with the manufacturer’s protocols.

### Oil Red O staining

3T3-L1 cells were fixed with 4% (v/v) paraformaldehyde (Abiowell, AWI0056b) at room temperature for 30 min. Cells were stained with 60% Oil Red O (Abiowell, AWI0580a) at room temperature for 30 min and then washed with PBS three times. The lipid droplets are examined and captured on camera under a light microscope (Zeiss, Germany). A 100% isopropanol solution was added to extract Oil Red O, and its concentration was then determined by measuring absorbance at 492 nm.

### Biochemical assessment

The serum levels of fasting plasma glucose, total cholesterol, high-density lipoprotein cholesterol, and low-density lipoprotein cholesterol were analyzed using a Hitachi 7600 automatic analyzer manufactured (Hitachi Ltd., Japan). The measurement of all indicators was conducted in a medical laboratory accredited under ISO 15189, under defined conditions.

### RNA sequencing

The established experimental techniques described were followed for the transcriptome sequencing at Genechem Biotechnology Co., Ltd. in Shanghai, China. The RNA of cells (shIL-33 versus shNC; *n* = 3 each) was extracted with TRIzol Reagent (Invitrogen, 15596026) 10 days after adipogenic induction. A total amount of 1 µg RNA per sample was used as input material for the RNA sample preparations. Sequencing libraries were generated using NEBNext® UltraTM RNA Library Prep Kit for Illumina® (New England Biolabs, Catalog #E7530) as follow and index codes were added to attribute sequences to each sample. Image data from high-throughput sequencers were converted into sequence data (reads) in fastq format by CASAVA base recognition. The raw data are then filtered. All subsequent analyses are of high quality based on clean data. The index of the reference genome was constructed using HISAT2 v2.0.5, and the paired end clean reads were compared with the reference genome using HISAT2 v2.0.5. DESeq2 software (1.16.1) was used to analyze the differential expression between the two comparison combinations (padj < 0.05,|log2FoldChange|>1). Statistical enrichment of differentially expressed genes in the KEGG pathway was analyzed using clusterProfiler (3.4.4) software.

### Immunofluorescence staining

The 3T3-L1 cells were cultured on glass coverslips with a density of 1 × 10^5^ cells per milliliter and induced to adipogenesis for 10 days. The samples underwent a washing step using phosphate-buffered saline (PBS), followed by fixation by 4% paraformaldehyde solution. Subsequently, the samples were permeabilized with a 0.5% Triton X-100 solution (Beyotime, C1048) in PBS at room temperature for 15 min, and then, blocked with 0.5% goat serum solution (Beyotime, C0265) for 1 h. Following this, the cells were incubated with rabbit monoclonal anti-β-catenin (Cell Signaling Technology, #8480, 1:100) at a temperature of 4℃ for an overnight period. The cells were then exposed to incubation with Alexa Fluor 488 goat anti-rabbit antibody (Thermo Fisher Scientific, Cat. No. A27034) after primary antibodies were removed and rinsed with phosphate-buffered saline (PBS). This incubation took place in a light-protected environment at ambient temperature for 1 h. Following the application of DAPI (Beyotime, C1006) staining and subsequent mounting, the acquisition of pictures was performed utilizing laser scanning confocal microscopy (Zeiss, Germany).

### Statistical analysis

Statistical analysis was performed using Prism software version 8.0 (GraphPad Software) and IBM SPSS Statistics 27. A non-parametric test was used for non-normally distributed data. Two-tailed Student’s t-tests and one-way ANOVA were used for normally distributed data when appropriate. All data in the figures are shown as the mean ± SEM. A P value < 0.05 signifies a statistically significant difference.

## Results

### IL-33 in visceral adipose tissue was upregulated in an HFD

**Fig. 1:**
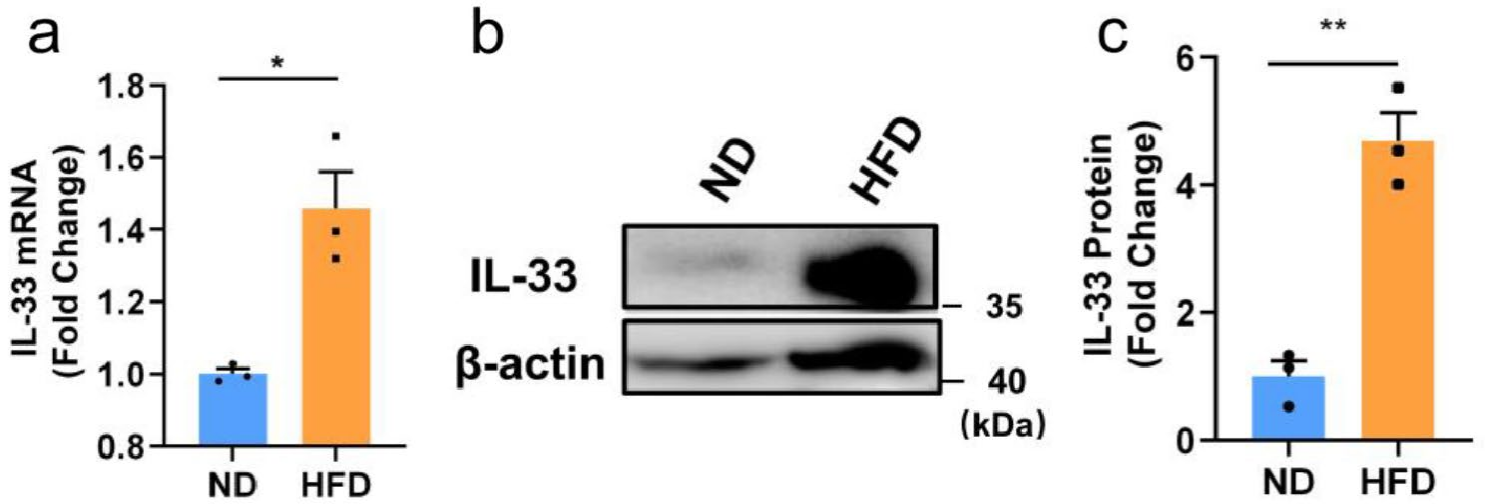
IL-33 level increased in the VAT of HFD mice. Mice were fed an HFD or ND for 8 weeks. (**a**) qPCR analysis of IL-33 mRNA expression in VAT from ND and HFD mice for 8 weeks. (**b** and **c**) Western blot analysis of IL-33 expression in VAT from ND and HFD mice for 8 weeks and densitometry quantification. The data are represented as mean ± SEM (*n* = 3). ^*^P < 0.05 and ^**^P < 0.01 versus the ND group. HFD, high-fat diet; ND, normal diet; IL, interleukin; qPCR, quantitative PCR; VAT, visceral adipose tissue

We first investigated the difference in the tissue level of IL-33 based on the obese mouse model of HFD. In the eighth week of feeding, the mouse model of obesity was successfully established (Fig. S1). After 8 weeks, no significant difference was found in the serum levels of IL-33 between the mice in the ND and HFD groups (Fig. S1). In contrast, as opposed to the ND group, the HFD group exhibited higher levels of IL-33 mRNA expression in visceral adipose tissue (VAT) after 8 weeks (Fig. 1a). Moreover, we also analyzed the expression levels of IL-33 protein in VAT. Western blot examination conducted in the eighth week of feeding revealed that the HFD group had considerably higher levels of IL-33 expression in viscera adipose tissue than the ND group (Fig. 1b and c). These results suggest that IL-33 may play an important role in the formation of obesity.

### Expression level of IL-33 was upregulated during the adipogenesis of 3T3-L1 cells

**Fig. 2:**
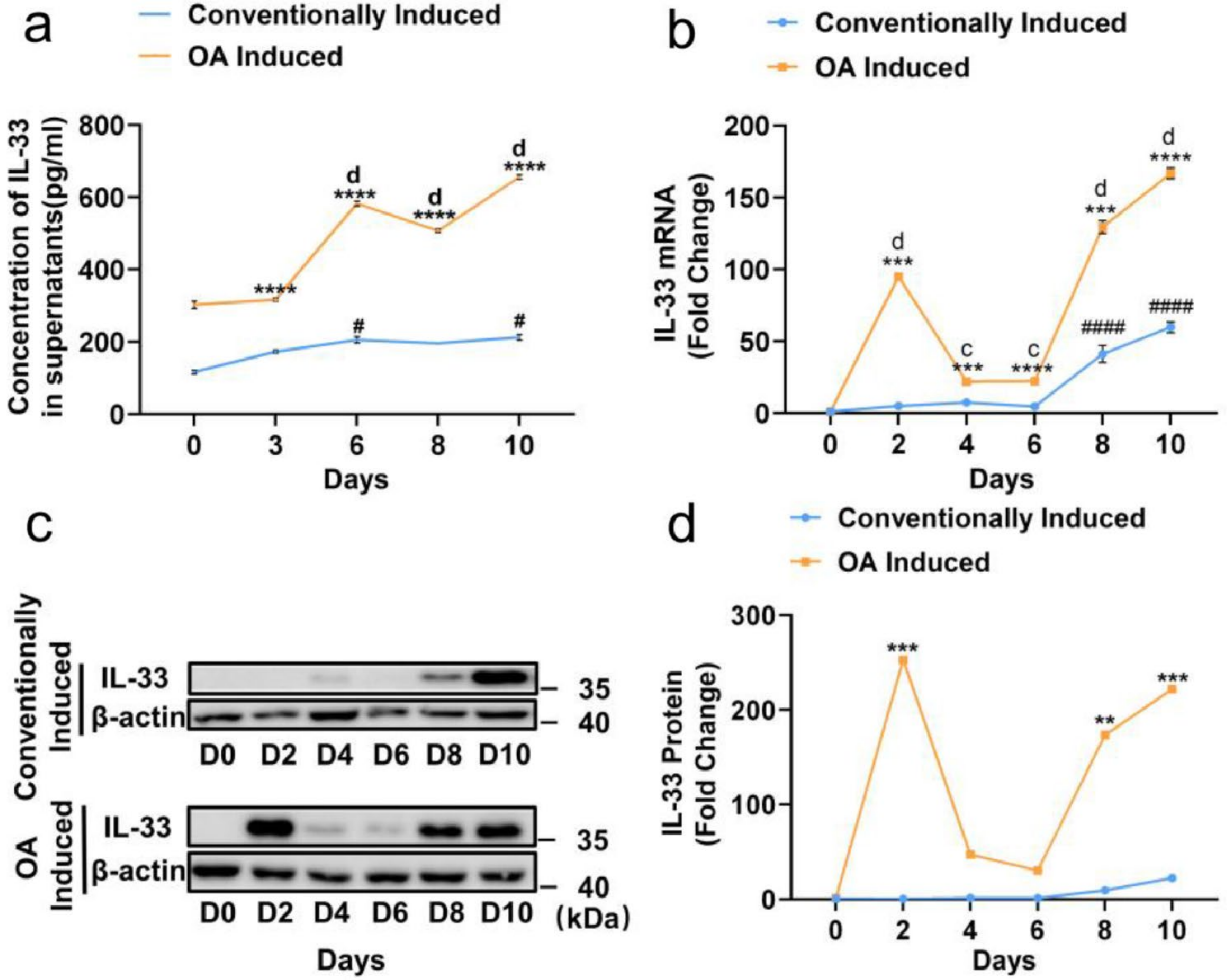
Levels of IL-33 increased during adipogenesis. 3T3-L1 cells were induced by conventionally adipogenic differentiation and OA-induced hyperadipogenic differentiation. (**a**) Secretion levels of IL-33 during adipogenesis of 3T3-L1 cells in conventional and hyperadipogenic groups were measured by ELISA. (**b**) RT-qPCR was conducted to evaluate the mRNA level of IL-33 following adipogenesis on days 0, 2, 4, 6, 8, and 10. (**c** and **d**) Protein level of IL-33 was measured by Western blot analysis. The data are represented as the mean ± SEM (*n* = 3). ^**^P < 0.01, ^***^P < 0.001, and ^****^P < 0.0001 versus the conventionally induced group at the same time points; ^#^*P* < 0.05 and ^####^*P* < 0.0001 versus day 0 in the conventionally induced group; ^c^*P*< 0.001 and ^d^*P*< 0.0001 versus day 0 in the OA-induced hyperadipogenic differentiation group

Next, in an effort to comprehend the function of IL-33 in the emergence of obesity, we examined whether preadipocytes secreted IL-33 and whether its expression altered during adipogenesis. 3T3-L1 preadipocytes were treated with cocktail induction. At the same time, we added OA to further simulate the hyperlipogenesis state. In the preadipocyte state, IL-33 was hardly expressed or secreted, but was significantly expressed and secreted after differentiation (Fig. 2a–d). ELISA revealed that the expression of IL-33 was induced in 3T3-L1 cells during adipogenesis in both the conventionally induced group and the OA-induced group (Fig. 2a). IL-33 levels increased continuously after day 6 during adipogenesis of 3T3-L1 cells in both the conventionally induced group and the OA-induced group (Fig. 2b–d). Moreover, the elevation of IL-33 was higher in the OA induced group than in the conventionally induced group (Fig. 2a and d). These findings suggested that IL-33 might play a significant role in adipogenesis.

### Inhibition of IL-33 expression promoted adipogenesis during preadipocyte differentiation

**Fig. 3:**
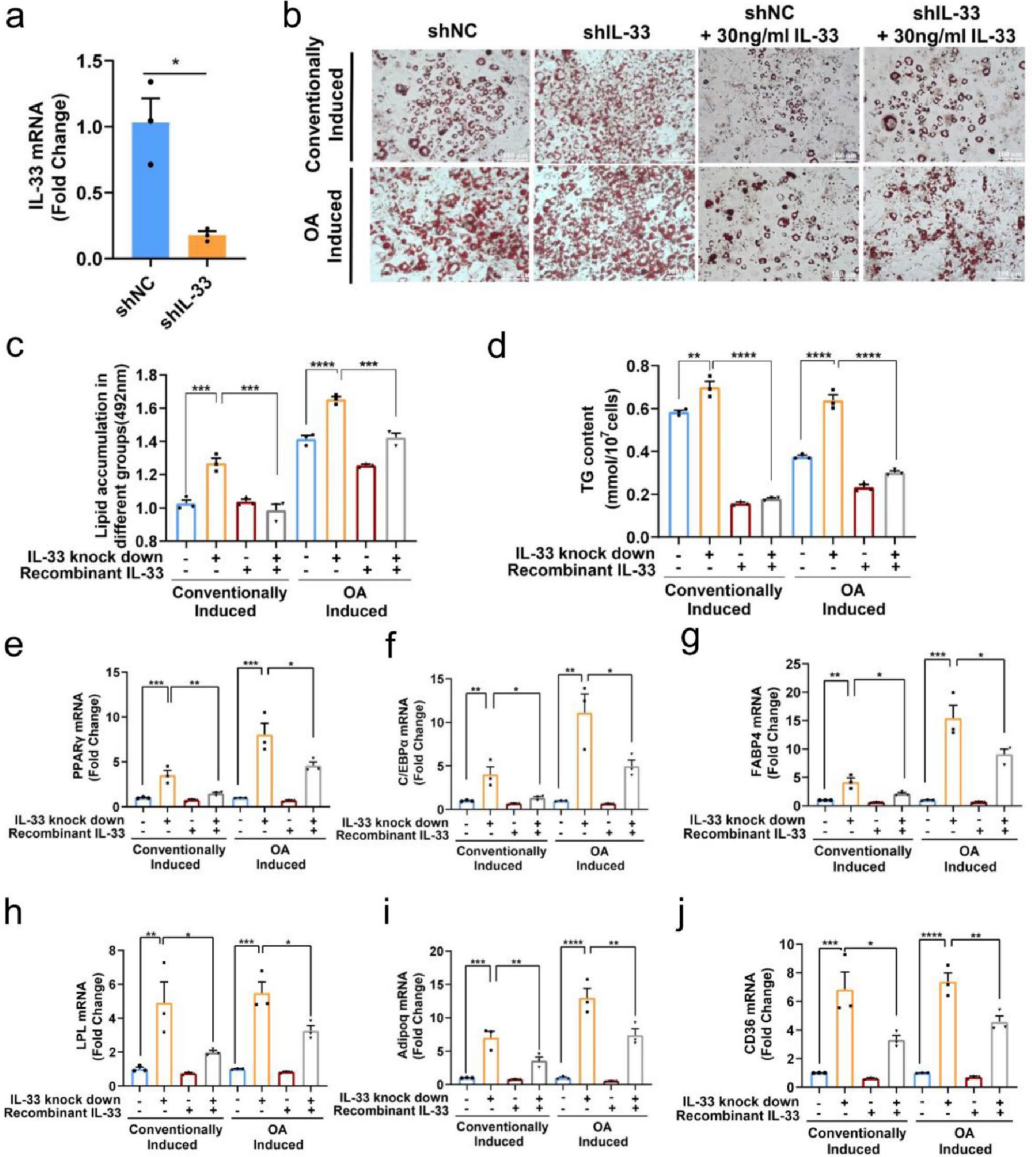
Knockdown of IL-33 promoted the adipogenesis of 3T3-L1 preadipocytes. Before adipogenesis, 3T3-L1 cells were transfected with IL-33 siRNA, and 72 h later, the cells were incubated for adipogenic differentiation and treated with OA for hyperadipogenic differentiation. Meanwhile, the cells were induced with 30 ng/mL recombinant IL-33. The cells after 10-day induction were tested. (**a**) The efficacy of IL-33 knockdown in 3T3-L1 cells was confirmed using RT-qPCR. (**b**) Oil Red O staining; scale bar = 100 μm. (**c**) Semi-quantitative detection of cells in different groups using Oil Red O staining (detection at 492 nm). (**d**) Cellular TG levels were quantified. (**e**–**j**) RT-qPCR was conducted to evaluate the expression of the adipogenic factors PPAR-γ, C/EBPα, FABP4, LPL, Adipoq and CD36 after 10-day induction. The data are represented as the mean ± SEM (*n* = 3). ^*^P < 0.05, ^**^P < 0.01, ^***^P < 0.001 and ^****^P < 0.0001. PPAR-γ, Peroxisome proliferator-activated receptor gamma; C/EBPα, CCAAT/enhancer-binding protein α; FABP4, Fatty acid binding protein 4; LPL, lipoprotein lipase; Adipoq, adiponectin; CD36, platelet glycoprotein 4

We examined the involvement of IL-33 in adipogenesis by lentivirus interference. In comparison to cells subjected to control siRNA treatment, the cells that underwent transfection with siRNA demonstrated a noteworthy reduction in the expression level of IL-33, as quantified using qPCR analysis (Fig. 3a). Then, the adipogenic differentiation ability of 3T3-L1 cells was compared after the conventional induction of adipogenesis and the induction of OA by the cocktail method. Our results demonstrated that the downregulation of IL-33 significantly increased the quantity of lipid droplets and the accumulation of TG (Fig. 3b–d). Additionally, the expression of the adipocyte differentiation marker genes PPAR-γ, C/EBPα, FABP4, LPL, Adipoq, and CD36 increased dramatically after IL-33 knockdown in 3T3-L1 preadipocytes (Fig. 3e–j). Moreover, the same results were also obtained in the OA-induced group (Fig. 3e–j). These results suggested that the inhibition of IL-33 expression during differentiation promoted adipogenesis.

We performed IL-33 recombinant protein supplementation experiments to further confirm the role of IL-33 in adipogenic differentiation. 30 ng/mL of IL-33 recombinant protein was added to the 3T3-L1 cells with IL-33 knockdown. The results demonstrated that the supplementation of IL-33 protein dramatically inhibited lipid accumulation and reduced intracellular TG content during differentiation, whether in the conventionally induced group or the OA-induced group (Fig. 3b–d). At the same time, the elevated transcription of adipogenic differentiation–related genes PPAR-γ, C/EBPα, FABP4, LPL, Adipoq, and CD36 due to IL-33 knockout was significantly inhibited by exogenous IL-33 protein (Fig. 3e–j). According to these findings, IL-33 knockdown promoted adipogenesis.

### IL-33 overexpression inhibited adipogenic differentiation

**Fig. 4:**
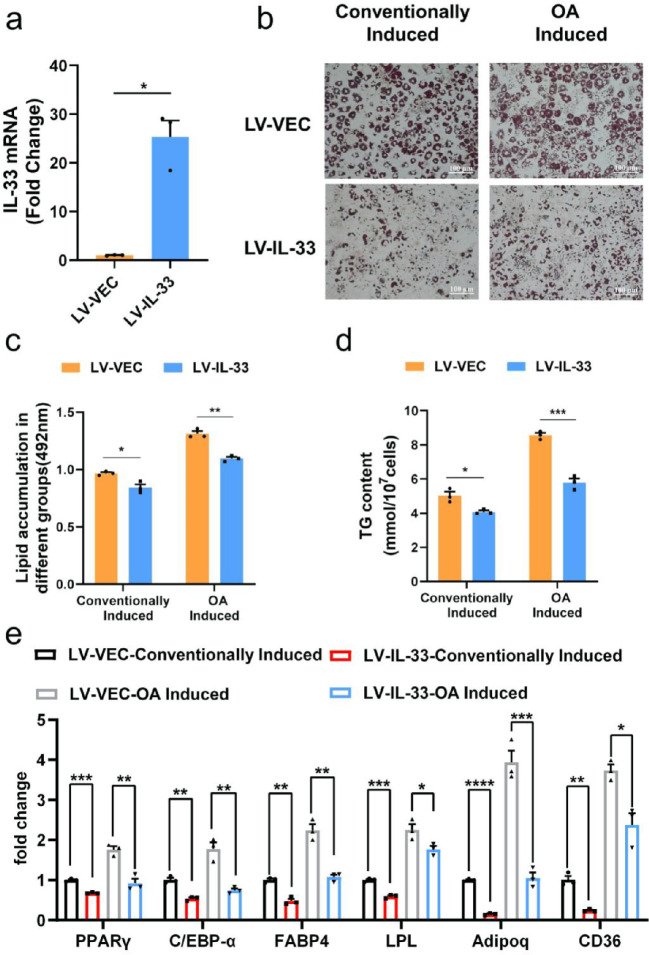
IL-33 overexpression inhibited adipogenesis in 3T3-L1 cells. 3T3-L1 cells were transfected with LV-IL-33 before adipogenic differentiation, and 72 h later, the cells were incubated for adipogenic differentiation and treated with OA for hyperadipogenic differentiation. (**a**) qPCR verified the efficiency of IL-33 overexpression in 3T3-L1 cells. (**b**) On day 10 of adipogenic differentiation, 3T3-L1 cells were stained with Oil Red O; scale bar = 100 μm. (**c**) Semi-quantitative detection of cells in different groups by Oil Red O staining (detection at 492 nm). (**d**) Cellular TG levels were measured after 10-day induction. (**e**) qPCR was conducted to evaluate the expression of the adipogenic factors PPAR-γ, C/EBPα, FABP4, LPL, Adipoq and CD36. The data are represented as the mean ± SEM (*n* = 3). ^*^P < 0.05, ^**^P < 0.01, ^***^P < 0.001, and ^****^P < 0.0001. PPAR-γ, Peroxisome proliferator-activated receptor gamma; C/EBPα, CCAAT/enhancer-binding protein α; FABP4, Fatty acid binding protein 4; LPL, lipoprotein lipase; Adipoq, adiponectin; CD36, platelet glycoprotein 4

We further verified the inhibitory impact of IL-33 on adipogenesis. IL-33 gene was overexpressed in 3T3-L1 cells by lentivirus (Fig. 4a). Then, the adipogenic differentiation ability of 3T3-L1 cells was compared after the conventional induction of adipogenesis and the induction of OA by the cocktail method. Our study revealed that the overexpression of IL-33 resulted in a significant reduction in the quantity of lipid droplets and the buildup of triglycerides (TG) (Fig. 4b–d). We performed qPCR experiments on individual genes to further validate the adipogenic differentiation levels. The expression of the adipogenesis-related genes PPAR-γ, C/EBPα, FABP4, LPL, Adipoq and CD36 were inhibited in IL-33-overexpressing cells (Fig. 4e). Moreover, the same results were also obtained in the OA-induced group (Fig. 4b–e). In summary, our results demonstrated that IL-33 inhibited adipogenesis during preadipocyte differentiation.

### IL-33 regulated PPAR signaling by suppressing the Wnt/β-catenin pathway

**Fig. 5:**
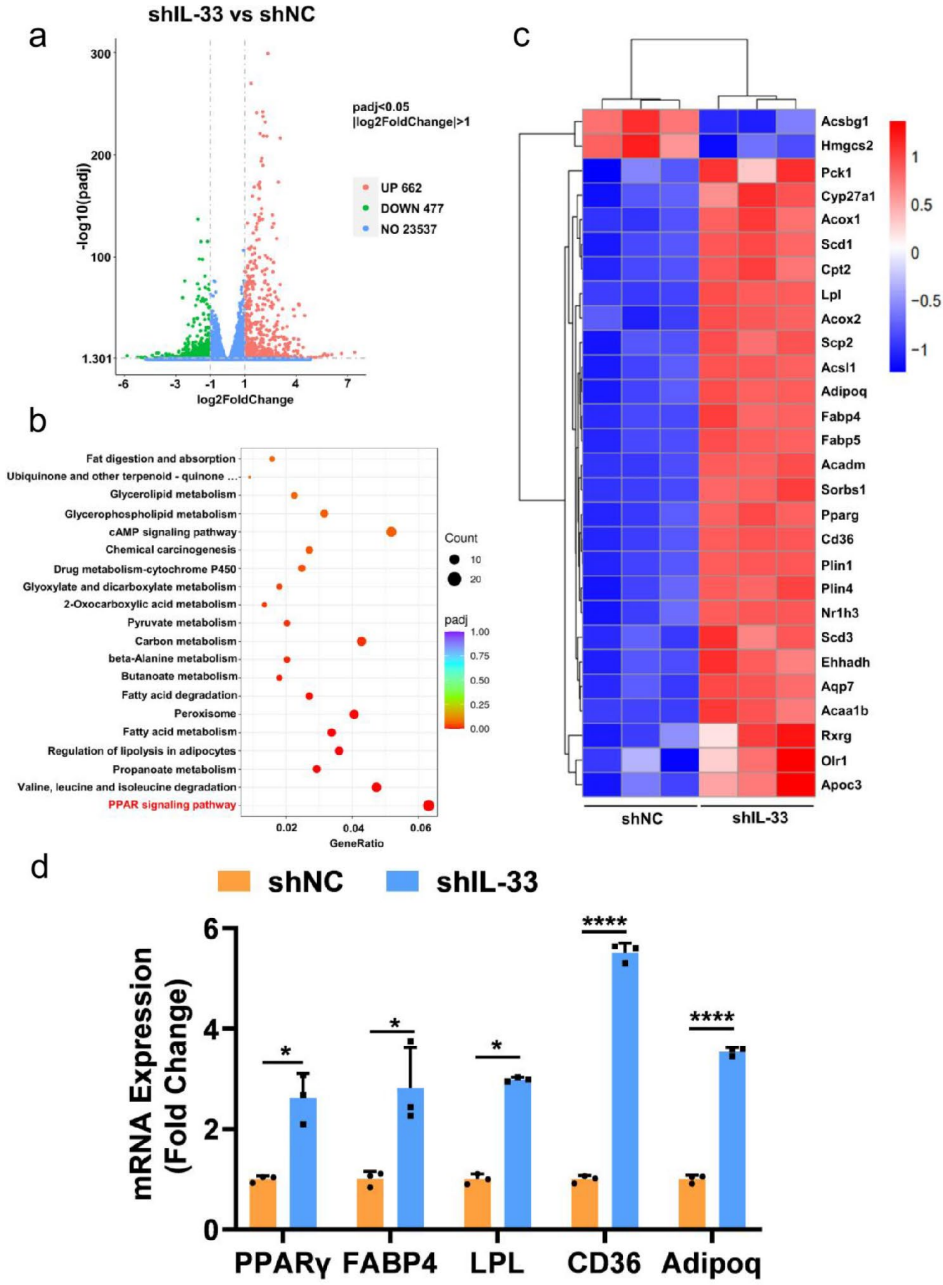
Knockdown of IL-33 promoted the PPAR signaling pathway. Following the induction of adipogenesis on day 10, 3T3-L1 cells with and without IL-33 knockdown were collected, and subjected to transcriptome profiling. (**a**) A scatterplot depicting the differential gene expression (DEG) values between the control cells and the IL-33-knockdown cells(fold change ≥ 1 and *P* ≤ 0.05). (**b**) The Kyoto Encyclopedia of Genes and Genomes (KEGG) analysis was performed on all differentially expressed genes (DEGs). (**c**) A heat map illustrating DEGs between the control cells and the cells with IL-33 knockdown. (**d**) Real-time qPCR analyses to validate the change in gene expression revealed by RNA sequence. The data are represented as mean ± SEM of three different samples (*n* = 3) from at least three independent experiments. ^*^P < 0.05 and ^****^P < 0.0001. PPAR-γ, Peroxisome proliferator-activated receptor gamma; FABP4, Fatty acid binding protein 4; LPL, lipoprotein lipase; Adipoq, adiponectin; CD36, platelet glycoprotein 4

Transcriptome sequencing was used to better investigate the particular mechanism of IL-33 on adipogenesis. The mRNAs extracted from IL-33 knockdown and control cells were sent for transcriptome sequencing after induced differentiation for 10 days. The findings indicated that there were an upregulation of 662 genes and a downregulation of 477 genes in the IL-33 knockdown group as compared to the control group (Fig. 5a). KEGG enrichment analysis showed that the PPAR signaling pathway was significantly enhanced in the IL-33 knockdown group, suggesting that the potential involvement of the PPAR family in the regulatory mechanisms of IL-33 during adipogenesis (Fig. 5b). Meanwhile, PPAR-γ downstream gene expression was increased (Fig. 5c). We performed qPCR experiments on individual genes to further validate the microarray results, focusing on PPAR signaling pathway–related genes including PPAR-γ, FABP4, LPL, CD36 and Adipoq (Fig. 5d); all of them were upregulated after IL-33 knockdown.

**Fig. 6:**
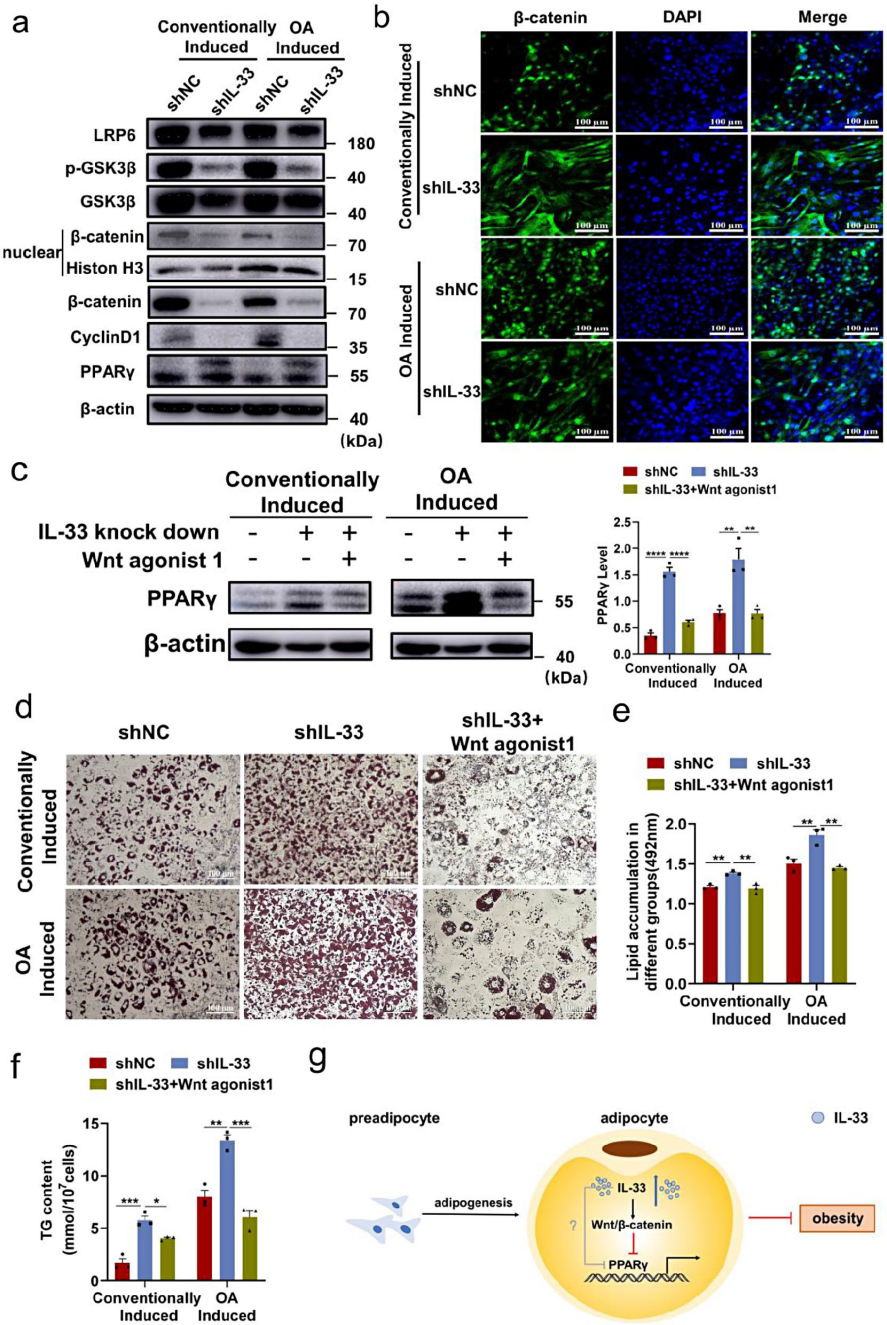
IL-33 was modulated in adipogenesis via the Wnt/β-catenin/PPAR-γ signaling pathway. 3T3-L1 cells were transfected with IL-33 siRNA before adipogenic differentiation, and 72 h later, the cells were incubated for adipogenic differentiation and treated with OA for hyperadipogenic differentiation. (**a**) Protein levels of LRP6, GSK3β, p-GSK3β, β-catenin, cyclin D1, PPAR-γ, H3, and β-actin were measured by Western blot analysis. (**b**) Expression of β-catenin was detected by indirect immunofluorescence staining with FITC-conjugated corresponding secondary antibodies (scale bar = 100 μm). DNA was stained with DAPI. (**c**–**g**) Cells were induced with Wnt agonist 1 (10µM) and collected on day 10. (**c**) Protein levels of PPAR-γ were measured by Western blot analysis. (**d**) Oil Red O staining was conducted after adipogenesis (scale bar = 100 μm). (**e**) Semi-quantitative detection of cells in different groups by Oil Red O staining (detection at 492 nm). (**f**) Determination of cellular TG levels. (**g**) Schematic models illustrating the role of IL-33 in inhibiting adipogenic differentiation in adipocytes. The data are represented as the mean ± SEM (*n* = 3).^*^P < 0.05, ^**^P < 0.01, ^***^P < 0.001, and ^****^P < 0.0001

One of the components implicated in the process of adipocyte differentiation is the Wnt/β-catenin signaling pathway, which has been observed to hinder adipogenesis by suppressing the expression of PPAR-γ [19]. Subsequently, we investigated the potential involvement of β-catenin in the induction of PPAR-γ expression by IL-33. The protein levels of β-catenin were seen to be decreased in 3T3-L1 cells with IL-33 knockdown, in comparison to cells treated with the vehicle (Fig. 6a). Confocal microscopy also demonstrated the nuclear transfer of β-catenin (Fig. 6b). After 10 days of adipogenic induction, β-catenin expression increased in the nuclear region after IL-33 knockdown (Fig. 6b). Besides, cyclin D1 levels decreased as the downstream of β-catenin (Fig. 6a). Our results further revealed that the levels of LRP6 and p-GSK3β decreased when IL-33 was knocked down in 3T3-L1 cells (Fig. 6a).

The effect of a specific Wnt agonist (Wnt agonist 1) was assessed to verify that IL-33 regulated adipogenesis by a Wnt/β-catenin/PPAR-γ signaling pathway. Wnt agonist 1 significantly reversed the effects of the increase in IL-33 knockdown–induced adipogenesis. The protein expression level of PPAR-γ markedly decreased in IL-33-knockdown cells treated with Wnt agonist 1 (Fig. 6c). Consistently, Wnt agonist 1 treatment of IL-33 knockdown cells resulted in a reduction in lipid droplet number and TG level (Fig. 6d–f). Moreover, the same results were also obtained in the OA-induced group (Fig. 6c and f). These results suggested that IL-33 regulated adipogenesis by mediating the Wnt/β-catenin/PPAR-γ signaling.

## Discussion

This study found that IL-33 expression increased with adipogenic differentiation and inhibited lipid formation. Further mechanistic studies showed that IL-33 regulation of adipogenesis was related to Wnt/β-catenin/PPAR-γ signaling. These studies defined the physiological role of IL-33 as a negative regulator of adipogenesis, thereby balancing the obesity microenvironment.

Some studies found that IL-33 improved obesity and metabolism [6, 20]. Hence, we explored how IL-33 played a role in the development of obesity. We found that the serum IL-33 levels in mice were not consistent with the elevated trend in VAT after 8 weeks of HFD, and no significant difference was found in serum IL-33 levels compared with those in the ND group. Therefore, we speculated two reasons for this. Firstly, obesity was progressive, ergo 8 weeks of HFD did not cause serious metabolic disorders. Secondly, these results further suggested the complexity of IL-33 distribution in the body and its role in obesity.

In obese mice, the role of exogenous IL-33 has been well established. Treatment of adipose tissue cultured with IL-33 decreased the expression of adipogenic genes and the size and number of lipid droplets [21]. In vitro, the 3T3-L1 cell model showed that compared with the induction group, 30 ng/mL IL-33 treatment reduced the number of lipid droplets and down-regulated the expression of PPAR-γ [6]. However, the relationship between endogenous IL-33 and adipogenesis has not been studied. Our study showed that IL-33 mRNA and protein increased mainly in the late stage of adipogenesis. Through IL-33 knockdown and IL-33 overexpression of 3T3-L1 preadipocytes, we found that IL-33 was related to the adipogenesis of 3T3-L1 cells. Our data provided additional evidence for the involvement of IL-33 in the adipogenesis of preadipocytes. It seems crucial that IL-33 expression is increased and maintained at a relatively high level during differentiation, as down-regulation of IL-33 can profoundly promote adipocyte differentiation at the cellular level.

To further demonstrate the contribution of IL-33 to the development of obesity, we added an OA treatment group. OA, an important fatty acid in the composition of human and other animal bodies, is widely found in animal and plant sources of cooking oil [22]. Studies have confirmed that the long-term intake of OA can cause obesity in experimental animals, and the use of OA treatment in vitro cell culture can also promote the intensity of adipogenic differentiation and increase lipid accumulation in 3T3-L1 cells [23]. Therefore, in this study, we used the OA induction group to promote hyperlipogenesis and thus simulate the obesity state. It is worth mentioning that IL-33 expression showed a sharp increase in the OA group at the early stage of differentiation (day2), which was not observed in the conventional group. It has been reported that the effect of OA on lipid differentiation-related genes was not obvious at the early stage of differentiation (day0-day3) [24], but could promote an adaptive response and enhance cell tolerance through increased cellular antioxidative capacity and thus protect 3T3-L1 cells against subsequent oxidative stress-related damage [25]. We speculate that the transient elevation of IL-33 may act as an effector of the antioxidant protection of OA on 3T3-L1 cells to a certain extent. This interesting phenomenon deserves further investigation.

Wnt/β-catenin signaling is an important regulator of mesenchymal cell fate determination [19]. In cultured mesenchymal stem cells, the Wnt/β-catenin signaling pathway inhibits adipogenesis [26]. Meanwhile, IL-33 has been shown to be involved in osteogenic differentiation of dental stem cells by activating Wnt/β-catenin signaling [18]. However, the role of IL-33 in adipogenesis has not been reported. Here, we demonstrate that preadipocyte derived IL-33 inhibits adipogenesis by activating the Wnt/β-catenin pathway. LRP6 is down-regulated when IL-33 is knocked down, suggesting that it might be a factor regulating IL-33’s participation in adipogenesis. LRP6 has been reported to limit obesity by regulating Wnt/β-catenin and subsequently inhibiting adipogenesis [27]. According to our data, IL-33 may play a role in adipogenesis via regulating LRP6 signaling.

β-catenin is a well-known negative regulator of PPAR-γ. Studies have shown that the classical Wnt/β-catenin signaling pathway inhibits adipogenesis by regulating PPAR-γ expression [16, 17]. We found that knocking down IL-33 down-regulates β-catenin and inhibits the Wnt/β-catenin signaling pathway. It is hypothesized that the persistence of IL-33 during late adipogenic differentiation upregulates β-catenin and, in this form, inhibits the PPAR-γ pathway and the downstream adipocyte phenotype. However, whether IL-33 can directly act on PPAR-γ remains to be further investigated. These two mechanisms may work synergistically and further inhibit adipogenesis.

There are still limitations in this research. Firstly, only verification at the cellular level was conducted on the effect of IL-33 in adipogenic differentiation of 3T3-L1 preadipocytes, while lacking further experimental evidence at the animal level. Secondly, the specific form of IL-33 regulation was not clear in this study, since IL-33 may be directly involved in transcription regulation of adipose differentiation as a nuclear factor, or play an autocrine or paracrine function as a cytokine [28]. These deserve further clarification.

In conclusion, the present study suggested that the expression of IL-33 surprisingly increased during adipogenesis and played an inhibitor role by regulating the Wnt/β-catenin/PPAR-γ signaling pathway. This study could provide new insight for further research on IL-33 as a new intervention target for metabolic disorders.

### Electronic supplementary material

Below is the link to the electronic supplementary material.

Supplementary Material 1

## Data Availability

The data sets supporting the conclusions of this article are included within the article.
