# Supplementary material for: IL-33 regulates adipogenesis via Wnt/β-catenin/PPAR-γ signaling pathway in preadipocytes
Source: J Transl Med. 2024 Apr 17;22:363. doi: 10.1186/s12967-024-05180-0 (PMC11022325; doi:10.1186/s12967-024-05180-0)
Supplement: Supplementary file 1 — Supplementary Material 1 [file 12967_2024_5180_MOESM1_ESM.docx]

**Supplementary Materials for**

IL-33 regulates adipogenesis via Wnt/β-catenin/PPAR-γ signaling pathway in preadipocytes

**Danning Xu****^1^, Siqi Zhuang^1^, Hongzhi Chen^2^, Mengjie Jiang^1^ , Ping Jiang^1^, Qian Wang^1^, Xuemei Wang^1^, Ruohong Chen^1^, Haoneng Tang^1*^, Lingli Tang^1*^**

^1^Department of Laboratory Medicine, The Second Xiangya Hospital, Central South University, Changsha, Hunan, China

^2^National Clinical Research Center for Metabolic Disease, Key Laboratory of Diabetes Immunology, Ministry of Education, Metabolic Syndrome Research Center, and Department of Metabolism & Endocrinology, The Second Xiangya Hospital, Central South University, Changsha, Hunan, China

**^*^Tang Haoneng and Tang Lingli contributed equally to this study**

**Corresponding authors:**

Haoneng Tang. **E-mail:** [505462@csu.edu.cn](mailto:505462@csu.edu.cn).

Lingli Tang. **E-mail**: [linglitang@csu.edu.cn](mailto:linglitang@csu.edu.cn).

**This PDF file includes:**

Table S1

Fig. S1

**Table S1. List of primers used for real-time quantitative PCR(qRT-PCR).**

| **Name** | **Forward(5’-3’)** | **Reverse(5’-3’)** |
| --- | --- | --- |
| **actin** | AGCCATGTACGTAGCCATCC | CTCTCAGCTGTGGTGGTGAA |
| **IL-33** | GCTGCGTCTGTTGACACATT | CACCTGGTCTTGCTCTTGGT |
| **PPARγ** | ATGTCTCACAATGCCATCAGG | TCTGGGTTCAGCTGGTCGAT |
| **C/EBPα** | CAAGAACAGCAACGAGTACCG | GTCACTGGTCAACTCCAGCAC |
| **FABP4** | AAGGTGAAGAGCATCATAACCCT | TCACGCCTTTCATAACACATTCC |
| **LPL**  **Adipoq**  **CD36** | TGGCGTAGCAGGAAGTCTGA  GCCTGGAGAAGCCGCTTATGTG  ATGGGCTGTGATCGGAACTG | TGCCTCCATTGGGATAAATGTC  TGCCAGTGCTGCCGTCATAATG  GTCTTCCCAATAAGCATGTCTCC |

**Supplementary Figure. S1**


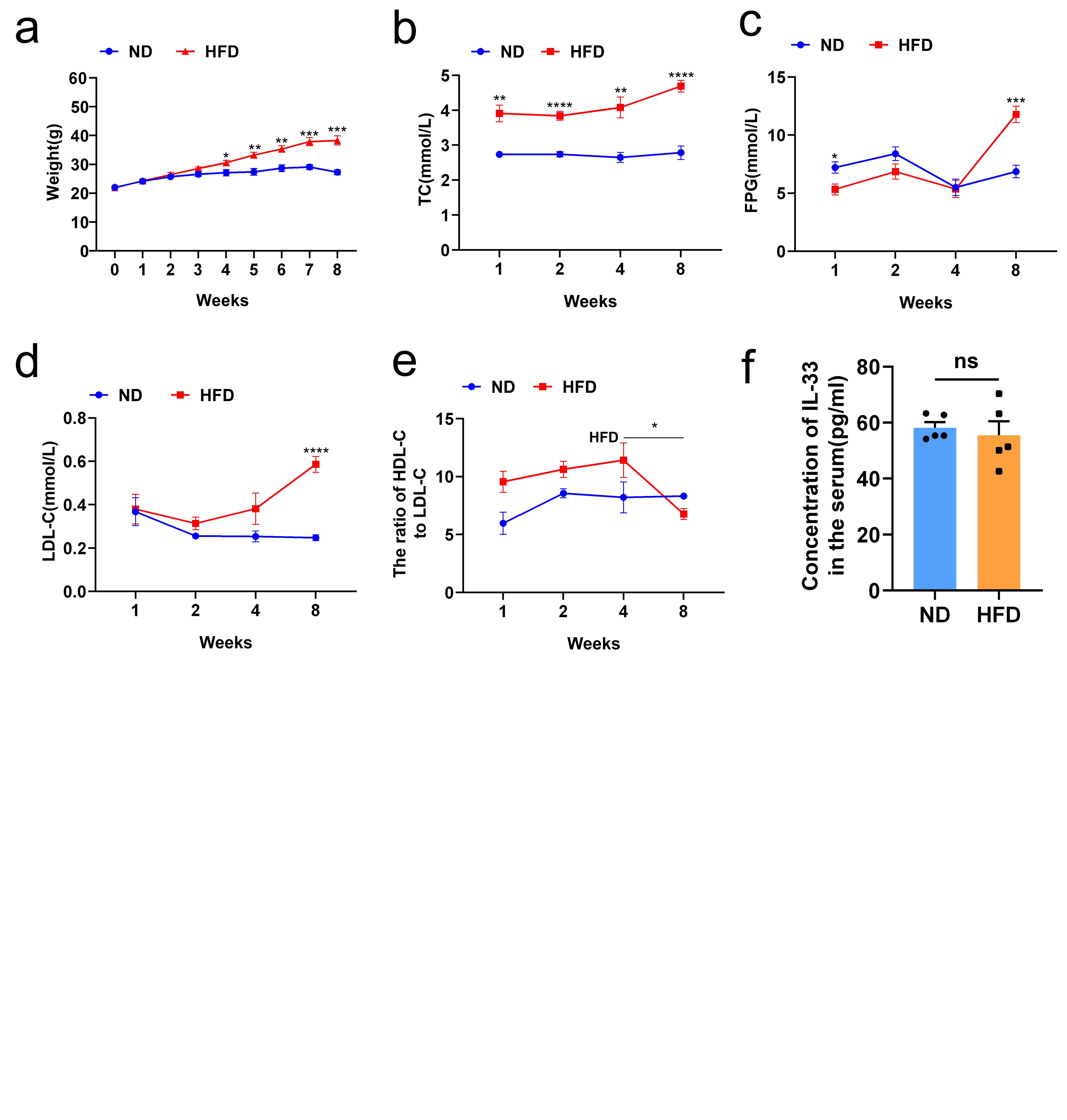


**Fig.S1. The model of obese mice fed high fat diet was established successfully and serum IL-33 levels were measured in eight-week-old mice.** Mice were fed with HFD or ND for 8 weeks. (a-e)the level of weights(a), TC (b), FPG(c), LDL-C(d) and HDL-C/LDL-C(e) of mice. (f) Serum IL-33 levels were detected by ELISA at 8 weeks. Data are represented as means ± SEM. n = 5 per group. *P< 0.05, **P < 0.01, ***P < 0.001 and ****P < 0.0001 versus the ND group. HFD, high-fat diet; ND, normal chow diet.
